# Communication, the centrosome and the immunological synapse

**DOI:** 10.1098/rstb.2013.0463

**Published:** 2014-09-05

**Authors:** Jane C. Stinchcombe, Gillian M. Griffiths

**Affiliations:** Cambridge Institute for Medical Research, University of Cambridge, Cambridge CB2 OXY, UK

**Keywords:** killing, centrosome, immunological synapse, cilia

## Abstract

Recent findings on the behaviour of the centrosome at the immunological synapse suggest a critical role for centrosome polarization in controlling the communication between immune cells required to generate an effective immune response. The features observed at the immunological synapse show parallels to centrosome (basal body) polarization seen in cilia and flagella, and the cellular communication that is now known to occur at all of these sites.

## 1. Directed cell-to-cell interaction in the immune system

The mammalian immune system is composed of a variety of cell types each specialized to combat a particular area of infection and disease. CD4 T cells, for example, act as ‘helper’ cells. On activation during an immune response, CD4 T cells release cytokines which can enhance the response of other immune cells. By contrast, cytolytic immune cells such as cytotoxic T-lymphocytes (CTL) and natural killer (NK) cells release lytic proteins which induce apoptosis and kill unwanted tumour or virally infected cells. To prevent aberrant killing, lytic proteins are stored within the immune cell in specialized ‘secretory’ lysosomes [1] (also called secretory or cytolytic granules) and only release their content on encountering an unhealthy cell.

**Figure 1.**
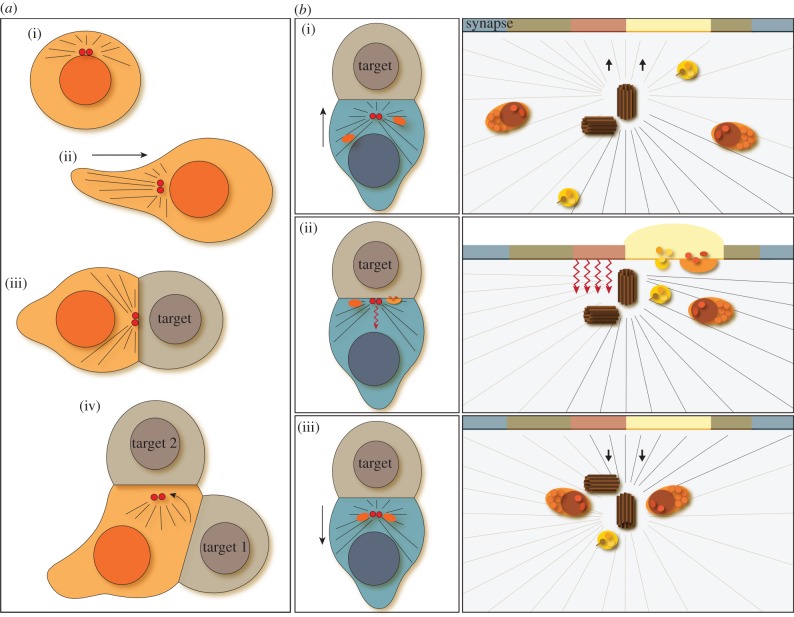
Properties of immune cell centrosomes interacting with target cells. (*a*) Centrosome positioning in T cells. In rounded naive or sedentary T lymphocytes (i), the centrosome (red) is close to the nucleus (dark orange) and microtubules (black) radiate from the cell centre towards the membrane. Centrosomes are located in the uropod at the back of the cell in migrating lymphocytes (ii), and rapidly polarize to the contact site synapse on interaction with a target (grey, target) (iii). On contact with further targets (iv), the centrosome retracts from the first target (1) and polarizes towards the second (2). Straight arrow in (ii) shows the direction of cell movement during migration. Curved arrow in (iv) shows repolarization of the centrosome on contact with a second target. (*b*) Centrosome polarization, secretion and signalling at different stages of immune cell interaction with targets. Low power cartoon (left) and corresponding medium power diagram (right) showing the different stages of immune cell (blue, cartoon; main cell, diagram) interaction with a target (grey, target, cartoon). On meeting a target (i), the immune cell centrosome (red, cartoon; brown, diagram) orientates towards the target and moves towards the contact site, reorganizing the microtubule network (black/grey lines) and microtubule-associated organelles, including secretory vesicles (yellow) and cytolytic secretory granules (orange). Signalling pathways are activated on cell : cell contact (red arrows). Tight centrosome polarization to the membrane (ii) aligns microtubules along the contact site and allows secretory organelles to access secretory sites at the synapse (pale yellow, diagram). Retraction of the centrosome (iii) removes the microtubule network and associated organelles from the contact site and prevents further secretion, terminating the functional response. Arrows indicate direction of centrosome movement. Membrane domains (or ‘supramolecular activation clusters’ (SMAC)) of the immunological synapse are indicated by colour bars in the diagram and show the integrin-containing peripheral ring (pSMAC, green) surrounding a TCR- and signalling protein-rich central domain (cSMAC, red) and a secretory domain (yellow) which, in lytic cells, forms beside the cSMAC and opposite an intracellular cleft (pale yellow, ii) (see also figure 2*a*).

**Figure 2.**
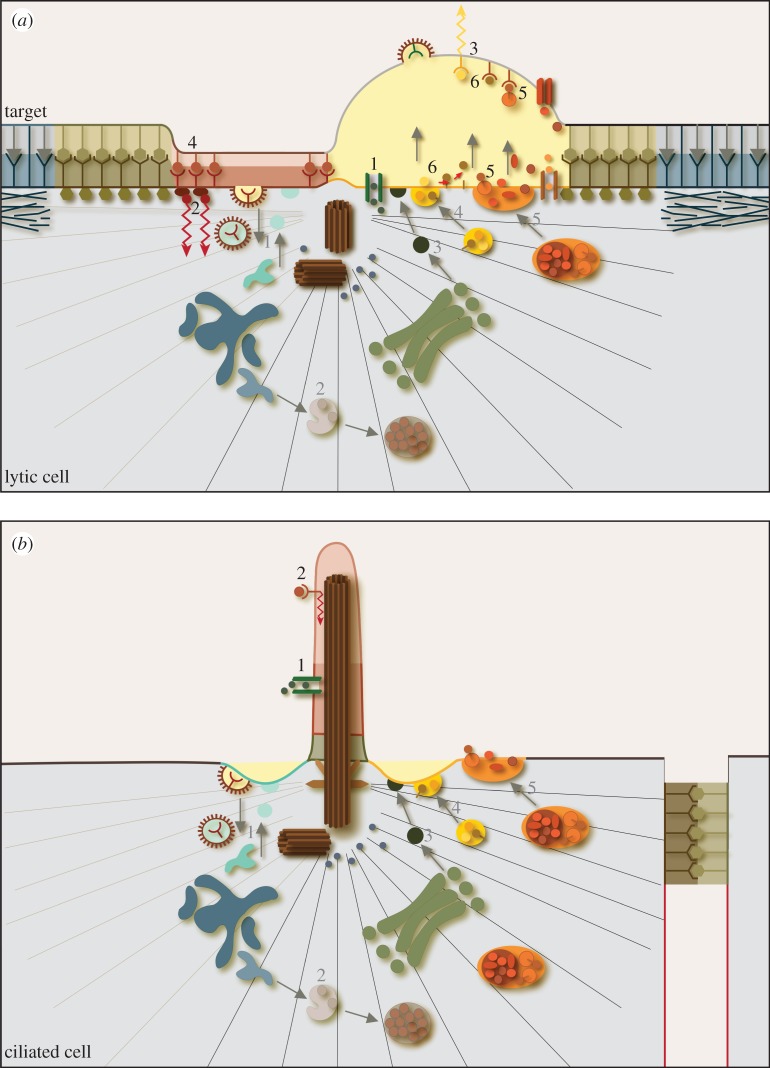
Morphological and functional similarities between the immunological synapse and cilia. Side views of the immunological synapse in CTL (*a*) and cilia region of a ciliated epithelial cell (*b*), showing centrosomes (brown), microtubules (black, grey lines) and associated organelles organized under the membrane. (*a*) Morphological features of the synapse include organization of the membrane into distinct ‘supramolecular activation cluster’ (SMAC) domains in which actin is present in a distal ring (dSMAC, blue) surrounding a peripheral ring of integrins (pSMAC, green) enclosing both a central (c)SMAC (red) containing TCR, and a secretory domain (yellow) opposite an intracellular cleft (pale yellow). The centrosome (brown) contacts the membrane between the cSMAC and secretory domain. Microtubule-associated organelles including the Golgi complex (light green) and biosynthetic vesicles (dark green), early and recycling endocytic compartments (blue), late endosomes and multivesicular bodies (grey/beige), constitutive secretory organelles (yellow) and regulated secretory lysosomes/lytic granules (orange) are present in the cytoplasm around the centrosome beneath the membrane. Signalling pathways (black numbers) include ion or protein channels (1) or membrane receptors (2,3). Membrane receptors can be activated at the cSMAC (2) or the target cell (3), by ligands (4–6) either present on opposing membranes (4), associated with released lysosomal vesicular content [1] (5), or released as free soluble proteins (6). Membrane trafficking pathways (grey numbers/arrows) include endocytic recycling (light blue, 1), degradative (grey, 2), biosynthetic (green, 3) and secretory (yellow/orange, 4,5) pathways. Secretory vesicles (yellow, 4) and cytolytic secretory organelles (orange, 5) are delivered to secretory sites at the cleft (pale yellow). (*b*) Morphological features of ciliated epithelia include (right hand side) separation of the apical surface (black membrane) from the basal (red membrane) by adherens junctions (sludge green) and (centre) organization of the surface membrane into distinct domains at and around the cilium, including the endocytic (turquoise) and secretory (yellow) regions of the cilia pocket at the cilium base, and the transition zone (green), inversin compartment (dark pink) and distal regions (light pink) of the cilium. The basal body (brown) sits at, and anchors, the base of the cilium. Microtubule-associated organelles including the Golgi complex (light green) and biosynthetic vesicles (dark green), endocytic recycling compartments (blue), late endosomes and multivesicular bodies (grey/brown), and constitutive (yellow) and regulated (orange) secretory organelles are all present around or near the centrosome at the cilia base. Signalling pathways (black numbers) include activation by extracellular ligands of ion or protein channels (1) or membrane receptors at the cilium membrane (2). Membrane trafficking pathways (grey numbers and arrows) include endocytic recycling (light blue, 1), degradative (dark blue/grey/beige, 2), biosynthetic (green, 3), and secretory (constitutive (yellow, 4) and regulated (orange, 5)) pathways.

The function of these and many other immune cells depends on intimate interactions between the immune cell and a second cell, usually termed the ‘antigen-presenting cell (APC)’ or ‘target’ (figure 1*a*(iii)). The nature of these interactions varies with respect to frequency, length and stability between different cells of the immune system. For example, cytokine production by CD4 T cells is often activated by interaction with dendritic cells. These interactions are stable, lasting for hours, and result in sustained cytokine production. Other interactions, for example those of CTL or NK cells with infected or tumour cells are fast, transient and sequential, and result in destruction of the unwanted ‘target’. Despite differences in duration, stability and outcome, most interactions share important structural and functional features and involve receptor-triggered recognition (for example by T-cell receptor (TCR) in T cells) accompanied by dramatic changes in protein organization and cell morphology. The membrane proteins across the cell : cell contact site reorganize to form a distinct topological rearrangement termed the ‘immunological synapse’ comprising membrane domains known as ‘supramolecular activation clusters’ (SMAC) (figures 1*b* and 2*a*). Synapse structures were first identified in CD4 T cells interacting with APC, in which a peripheral ring of T-cell integrin proteins (pSMAC) was found to surround a central (cSMAC) region that accumulated TCR and other signalling proteins [2,3]. An outer distal (dSMAC) ring, rich in actin and actin-associated proteins, has also been identified. Similar basic structures have since been identified at the contact site between many immune cells and their APCs although there are cell type-specific differences. For example, synapses formed between CTL and unhealthy target cells contain an additional central ‘secretory’ domain. This appears opposite a space between the two cells termed the secretory cleft into which lytic proteins are released [4] (figure 2*a*). It is thought that this sort of membrane organization allows cell-to-cell communication including exchange of signals, material and information between the two cells. Morphological changes involve polarization of the immune cell, in particular, orientation of the cytoskeleton towards the target cell [5–7] (figure 1*a*(iii)). Key to many of these changes, both at the membrane and within the cell, is polarization of the centrosome to the immunological synapse.

## 2. The polarized centrosome

Centrosome positioning varies in T cells (figure 1*a*). In migratory T cells, the centrosome is in the uropod, slightly distant from the nucleus (e.g. figure 1*a*(ii)). This contrasts with most migratory cells which move with the centrosome in front of the nucleus. Upon contact with a target, the centrosome begins to polarize towards the synapse (figure 1*a*(iii)). Recent studies suggest that polarization may be biphasic, with a fast initial ‘polarization’ phase during which the centrosome moves from the uropod round the nucleus to face the target, and a second slower ‘docking’ phase as it moves up to the synapse at the contact site [8]. Only the initial phase seems to occur when TCR signalling is compromised [9]. Live cell imaging has shown that the centrosome can oscillate back and forth at the membrane and may retract and abort completely, moving back into the cell body or polarizing to a different target [10]. At the end of the interaction the centrosome either repolarizes to a second target (figure 1*a*(iv)), moves back to its migratory position behind the nucleus in the uropod (figure 1*a*(ii)) or, in static cells, sits next to the nucleus (figure 1*a*(i)). Full retraction of the centrosome back into the cell body from the cell surface may be important in terminating the response at or before cell separation (see §7 and figure 1*b*).

The movement of the centrosome across almost the entire length of the migrating T cell is far more dramatic than that seen in other cell types where the centrosome may only polarize from one side of the nucleus to the other [11]. Very tight polarization of the centrosome to the plasma membrane at the synapse was first identified in studies on CTL and subsequently observed by electron microscopy (EM) for other lytic cells [12–15]. A time course of EM images suggests that the tight polarization process may take longer in CD4 T cells and only appears in samples fixed after 2–4 h of interaction, the time of peak cytokine secretion [14].

## 3. The centrosome as the master regulator

The importance of centrosome movement towards the contact site for immune cell function was first recognized in early studies showing that centrosome polarization towards the point of membrane contact occurred during target killing [5–7]. The centrosome was subsequently found to move right up to the contact site and associate with the membrane at a specific point between the region of TCR clustering and the sites of secretory granule docking and secretion [12,13]. These observations demonstrated a role for centrosome polarization in granule delivery to the secretory cleft since this reorganizes the microtubule network to lie parallel to the membrane under the secretory sites, thus directing and delivering the granules to the secretory sites during minus-end directed movement along the polarized microtubules [12,13] (figure 1*b*(i–ii)).

Other insights into centrosome polarization to the synapse have come from studies on B cells in which the centrosome was ablated [16,17]. B cells also secrete lysosomal content on interaction with APCs, and proteases released from the lysosomes are involved in extracting antigens from the target cell membranes. B cells lacking centrosomes showed no reorganization of their microtubule cytoskeleton, no movement of microtubule-organizing centre (MTOC)-associated organelles towards the target and no release of lysosomal content. Furthermore, lysosomes showed no directed movement either towards the contact site or the site of the ablated centrosome. Thus, the centrosome itself is required for polarized secretion in B cells.

Centrosome polarization directs many events at the synapse. MTOC-associated organelles including the Golgi complex, which processes newly synthesized proteins, and endocytic recycling compartments, responsible for downregulation of membrane proteins including TCR and associated proteins LAT and Lck [18–20], are focused at the immunological synapse when the centrosome is at the membrane (figure 2*a*). This means that centrosome polarization can effectively control communication at the synapse, with signalling, secretion and recycling effectively activated (‘switched on’) when the centrosome is in contact with the plasma membrane (figure 1*b*(ii)) and ‘switched off’ when retracted (figure 1*b*(i,iii)). This seems likely to be particularly relevant for controlling secretion from cytolytic cells because mutations which allow centrosome polarization close to, but not at, the plasma membrane, prevent secretion [9].

Centrosome polarization may not be required for secretion of all cytokines from CD4 T cells [21]. A recent study suggested cytokine release from CD4 T cells can occur in the absence of centrosome polarization [22]. Silencing of cdc42 at the site of secretion did not affect centrosome polarization but did prevent release of IL2 and IFN-γ, two cytokines that have been shown to polarize with the centrosome [23]. It was proposed that this might be due to loss of actin clearance from the site of release as cdc42 is required for actin remodelling at secretory sites.

## 4. Morphological similarities between centrosome polarization sites at the immunological synapse and cilia

Tight centrosome polarization right up to the membrane is unusual but is also seen in cells with cilia and flagella. Although cilia are much more stable than the transient synapses formed by immune cells, there are many morphological similarities between these structures (summarized in figure 2). Intriguingly, primary cilia and flagella are often embedded within invaginations of the cell membrane, termed the cilia/flagella pocket [24,25]. This pocket bears similarities to the secretory clefts which form opposite polarized centrosomes of immune synapses [4]. Curiously, the membrane of immune cells can form membrane bumps and protrusions that project into these clefts above the centrosome [13,26]. Although these may appear to parallel cilia structures they lack axonemes and their significance is unclear.

Evidence is now emerging that suggests that, like the synapse (figure 2*a*), the ciliary sheath and the plasma membrane surrounding the polarized centrosome of cilia and flagella (called basal body) is organized into distinct functional domains [27] (figure 2*b*). The transition zone acts as a selective barrier between the cell body and the cilium [27–29], while signalling, signal transduction and protein release occur within the cilium itself [30–33]. Thus, like the synapse of immune cells, the membrane surrounding polarized basal bodies at sites of cilia formation also contains distinct domains devoted to adhesion, signalling and secretion.

## 5. Molecular and functional similarities between cilia and the synapse

Several proteins required for ciliogenesis have also been shown to be involved in formation of the immunological synapse. Correct positioning of the basal body during ciliogenesis is known to involve polarity proteins including dishevelled (Dvl), Par3, Par6 and atypical protein kinase C proteins (aPKCs) [34,35]. Many of these have now been shown to be involved at different stages in T-cell polarity, with Par6 and the aPKC isoform aPKC-ζ involved during T-cell migration and detection of target cells [36], while Par3, Scribble and Discs large (Dlg) show polarized front-back and lateral distributions during interactions of T cells with APCs [37,38], and aPKC-ζ enriches with Par3 at the synapse [39].

Of particular interest, ‘cilia’ proteins have now been shown to be present in events at the immunological synapse. The intraflagellar transport (IFT) components, IFT88 and IFT20, first identified as part of the complex transporting material through cilia and flagella (reviewed in [40]), have been implicated in TCR recycling at the synapse [41]. Roles for IFT proteins outside the cilium in ciliated cells had previously been described. For example, IFT20 is involved in vesicular transport between the Golgi complex and the base of the growing/mature cilium [42] and in some ciliated cell types, such as ciliated retinal cells, appears exclusively localized to Golgi-associated vesicle transport pathways [43]. IFT88, although primarily involved in transport within the cilium, is also found associated with basal bodies at early stages of ciliogenesis [43] and is required for correct positioning and orientation of developing cilia within the apical membranes of epithelia [44]. The observations in immune cells were the first demonstration of a role for ciliary proteins in cells that do not make cilia. The involvement of IFT88 in immune cells is particularly intriguing given the role of the centrosome in positioning organelles at both the synapse and cilium. IFT20 has since been identified in other non-ciliated cells such as retinal neurons, where several IFT proteins localize to the dendrites [45]. Recently, the ciliary membrane protein inversin/nephrocystin-2 was shown to be required for the polarity and directional migration of fibroblasts [46]. Thus, there is increasing evidence of ciliary proteins playing more general roles in pathways outside the cilium (see also [47]).

As at the immunological synapse, secretion can also be focused at cilia (figure 2*b*). Unicellular paramecia release their specialized storage organelles (trichocysts) at distinct exocytic secretory domains at the base of cilia [48]. Membrane vesicles have been shown to be released from the cilium of *Chlamydomonas* [49]. These ‘ectosomes’ contain proteases required for cell wall breakdown during reproduction and provide a remarkable parallel to the release of lytic and lysosomal proteases from secretory lysosomes at the immunological synapse.

Signal transduction is now recognized as a major function of primary cilia, with recent reports suggesting that primary cilia are specialized calcium-signalling organelles [50]. Several signalling pathways have been identified in cilia including Hedgehog (Hh), Wnt, Notch, mTOR and receptor tyrosine kinase pathways (reviewed in [30–33]). A primary role for cilia in signalling is in cell-to-cell communication and information exchange. Thus, signalling in cilia parallels that in immune cells, where signalling is involved in, and results from, interaction of immune cells with targets. Cilia project from the cell surface allowing them to transduce signals between the extracellular environment and the cell body. Components of the signalling pathways (including membrane receptors, signal transducers (including ion channels) and effector proteins) are localized to the cilia membrane which allows them to detect and respond to extracellular ligands and/or changes in the external environment [30–33] (figure 2*b*). Signal reception and transduction may be molecular, via detection of chemical changes (e.g. for unicellular organisms) or binding of ligands to receptors on the cilia membrane (e.g. binding of Hh to Patched (Ptch) [51–53]) or at the cilium base (e.g. Wnt binding to Frizzled) and activate downstream signalling pathways within the cilium and/or the cell body. Alternatively, extracellular signals may be mechanical, mediated by bending forces on the cilia membrane which activate calcium fluxes and are driven, for example, by fluid flow in the surrounding milieu [54,55] (e.g. for renal tubule cells of the kidney). In addition, cilia generate signals which act locally or at a distance, for example by cleavage of peptide domains (e.g. proteolytic processing of polycystin-1 in the kidney duct) or shedding of membrane [49] from the cilia surface.

Hh pathways are involved in regulating a number of developmental processes. Many key steps involved in Hh signalling occur within the primary cilium, and movement and exchange of proteins between the cilia and the cell body is critical for controlling Hh signalling pathways (reviewed, for example in [51–53]). Binding of extracellular Hh to Ptch on the cilia surface causes translocation of Ptch out of the cilium and movement of the transmembrane protein Smoothened (Smo) into it. Relocation of Smo to the primary cilium is required for translocation of Gli pathway activators to the nucleus and initiation of Gli-mediated transcription of target genes. It has been known for some time that Hh signalling is required during T cell development in the thymus [56]. What has only recently emerged is that TCR signalling at the immunological synapse triggers Hh signalling within T cells [57]. The Hh pathway plays an important functional role in CTL since cells in which Smo is deleted show impaired target cell killing. How does this happen? It takes 4–5 days for CTL to be generated from naive T cells, and TCR signalling is required to trigger small, naive T cells to produce the cytolytic secretory granules and dynamic cytoskeleton required for delivery of these secretory granules to the synapse. Although granules develop normally after Smo deletion, Rac1, required for actin remodelling and centrosome polarization [58–60], fails to upregulate. In this way, Hh signalling triggered by TCR at the synapse controls the ability of the centrosome to polarize to the synapse which is required for CTL to destroy target cells.

## 6. Polarized movement of centrosomes to and from the cell surface in immune and cilliated cells

The details of the mechanics controlling centrosome movement in immune cells are gradually emerging from a number of different studies. IQGAP1 forms part of a complex of proteins linking the plus ends of microtubules to the actin cortex [61,62]. Our early investigations suggested that IQGAP might link the movement of the centrosome to the reorganization of actin at the synapse [12]. This was supported by the finding that dynein associated with the outer ring of actin at the synapse, suggesting dynein might play a role in pulling the centrosome towards the synapse [10]. More recently, the centrosomal protein CK1d has been shown to be involved since its depletion leads to loss of centrosome polarization [63]. Intriguingly, CK1d phosphorylates the microtubule plus-end binding protein EB1, an activity that seems to be required for centrosome translocation, leading to the proposal that Ck1d plays a role in centrosome polarization in T cells by increasing microtubule growth speeds. The most recent observations on centrosome polarization also implicate microtubule dynamics in bringing the centrosome forward to the synapse [8]. This study suggested that the ‘pioneer’ microtubules seen in migrating cells [64] might also be present and tethered at the synapse and shrink as the centrosome moves forward, raising the possibility that centrosome polarization occurs by a process of end-on capture-shrinkage.

A growing body of evidence suggests formins are also involved in the mechanisms and direction of centrosome movement (reviewed in [65]). Formins are Rho-family GTPase effectors which act as linear actin nucleators and have roles including stabilization of microtubules. TCR activation induces the formins INF2, DIA1 and FMNL1 to form an array of stable, detyrosinated, glutamated (Glu) microtubules around the centrosome. It is thought that these disrupt the steady-state organization of the microtubule network and that this results in pushing forces which drive and direct the centrosome forwards towards the target APC. These arrays of Glu-microtubules may also be involved in positioning and stabilizing the polarized cytoskeleton at the synapse during target cell interaction.

Taken together, these data support the idea that actin and microtubule dynamics at the synapse play a role in generating the forces to pull the centrosome up to the synapse. Less is known about movement of the centriole to the surface during ciliogenesis. Early studies on primary cilia formation by Sorokin [66–68] suggested mother centrioles docked with membrane vesicles while still in the cell body. These vesicles coalesced to form vacuoles and the whole structure relocated to the surface. Axoneme extension and primary cilia growth was thought to initiate while the centrosome was still within the body and/or during transit to the surface. In other cells and tissues, centrosomes may interact directly with the plasma membrane at the cell surface, with transport of the centrosome mediated by forces generated by the cytoskeleton, possibly as has been proposed for immune cells (reviewed in [69,70]). It is not clear if the apparent differences in membrane association site reflect mechanistic differences between distinct cell or cilia types, for example primary cilia versus cilia on terminally differentiated cells, or whether different components of a single common pathway have been identified in distinct systems. Comparisons between centrosome behaviour during ciliogenesis and the mechanisms reported for immune cells may help elucidate the processes involved in both pathways in the future.

## 7. Centrosome retraction

At the end of interaction of an immune cell with a target, the centrosome retracts back from the surface into the cell body (figure 1*b*(iii)) and the domain organization of the synapse membrane proteins disperses as the two cells separate. Retraction of the basal body is also seen during downregulation of cilia and flagella in response to changes in the extracellular environment, for example tissue damage, release of stress or changes in nutritional supply. For immune cells, retraction allows repolarization to further targets or return to a migratory phase (figure 1*a*(ii, iv)), while for ciliated cells it permits re-entry into the cell cycle and cell division. In the unicellulate *Chlamydomonas*, flagella are resorbed under stress conditions by severing between the basal body and transition zone by the microtubule ATPase, Katanin p60 [71–73], which is thought to liberate basal bodies to return to the cell body for mitosis. In mammalian cells, control is regulated by displacement of TTBK2 and MARK4 kinases and rebinding of CP110, Cep97, Kif24 and Trichoplein (reviewed in [74]). Little is known about the mechanisms governing retraction of the centrosome from the synapse since it is not yet known if or how the centrosome ‘docks’ at the plasma membrane. What is clear is that in immune cells, as in cilia, tight association with the plasma membrane appears to be able to regulate function at the immunological synapse.

## 8. Differences in the role of centrosome polarization at the synapse and the cilium

While cilia and immune synapses share common features, there are also notable differences in centrosome behaviour at the membrane. Ciliary basal bodies dock with the membrane with the mother centriole in front of the daughter and associated via the distal appendages of the mother centriole (figure 2*b*). On docking, distal end microtubules elongate from the mother centriole to form the axoneme of the cilium, and the basal body is anchored at the membrane, forming a stable structure. By contrast, immune cell centrosomes remain associated with the synapse only transiently. As CTL can kill targets rapidly (within 5 min of interaction) while moving between multiple targets [75], the time of centrosome interaction with the plasma membrane must be brief. To date, there is no evidence of cilia formation upon polarization of centrosomes to synapses [76,77]. It is not yet known whether this is because they lack components required for cilia formation, ciliogenesis is regulated or inhibited, or simply because centrosome association with the plasma membrane is too transient to allow ciliogenesis to be initiated.

## 9. Origins and evolutionary significance of centrosome polarization in immune cells

Cilia and flagella are found on cells across phyla and are of particular functional importance to unicellulates. Furthermore, although not all cells and tissues require cilia or flagella for their steady-state ‘normal’ function, most can produce primary cilia under particular conditions, for example nutritional or environmental stress. Thus, they are evolutionarily primitive and widely conserved [78]. The fact that immune cells show centrosome polarization and share similarities with cilia on polarization but do not appear to make cilia is therefore particularly striking. This has led to the proposal that haemopoietic cells might exploit mechanisms used for primary ciliogenesis to orientate cell polarity, trafficking pathways, signalling processes and information towards target cells on target cell encounter [12,26].

The absence of cilia in immune cells is also unusual because there is a correlation between the presence of a centrosome and the capacity to form cilia or flagella, and most cell types which lack flagella or cilia also lack centrioles. For example, unciliated myotubes lose their centrosomes during differentiation [79], while cilia are not observed on cells/species with alternative microtubule-organizing structures, for example *Dictyostelium* and *Saccharomyces cerevisiae* [78]. A few specialized cell types do appear to retain polarized centrosomes in the absence of cilia. Differentiated enterocytes show permanently polarized centrosomes at the apical surface but lose their cilia as embryogenesis and tissue differentiation progress [80]. The centrosome remains at the surface associated with a vestigial cilium remnant but the adult tissue entirely lacks mature cilia, suggesting downregulation or loss of cilia mechanisms with development and differentiation. Other specialized tissues have functionally modified cilia or flagella, differentiated for particular roles by accentuation or loss of specific cilia components. Transient centrosome polarization in haemopoietic cells could therefore be seen as another form of extreme cilia specialization, in this case without production of the cilium, and/or of downregulation of ciliogenesis as a result of the fully differentiated state. The identification of ‘ciliogenesis’ proteins such as IFT and Hh [41,57] in immune cells supports this idea and suggests that at least some mechanistic proteins are conserved within immune cells and required for their function. Further studies to determine whether additional ‘ciliogenesis’ or ‘ciliary’ proteins are present and/or play roles in immune cell function, and vice versa, should shed more light on the relationship between ciliogenesis and immune cell centrosome polarization.

## 10. Concluding remarks

Centrosome polarization to the immunological synapse is important for information exchange between cells of the immune system in order to generate an effective immune response. Morphological and functional parallels exist between information exchange at the synapse and events at cilia and flagella. Increasingly, data show that the two systems also share proteins identified as playing roles in centrosome behaviour in one or both systems, raising the possibility that they also share mechanisms relating to centrosome polarization and its associated functions, and suggesting evolutionary links. The fact that ‘ciliary’ proteins are now known to be present and/or function in non-cilia pathways and/or different cell types lacking cilia suggests that molecules present in both systems may have even more widespread roles and that several ‘cilia’ proteins may turn out to function more universally.
